# Effectiveness of SmartMoms, a Novel eHealth Intervention for Management of Gestational Weight Gain: Randomized Controlled Pilot Trial

**DOI:** 10.2196/mhealth.8228

**Published:** 2017-09-13

**Authors:** Leanne M Redman, L. Anne Gilmore, Jeffrey Breaux, Diana M Thomas, Karen Elkind-Hirsch, Tiffany Stewart, Daniel S Hsia, Jeffrey Burton, John W Apolzan, Loren E Cain, Abby D Altazan, Shelly Ragusa, Heather Brady, Allison Davis, J. Mick Tilford, Elizabeth F Sutton, Corby K Martin

**Affiliations:** ^1^ Pennington Biomedical Research Center Baton Rouge, LA United States; ^2^ Woman's Hospital Baton Rouge, LA United States; ^3^ United States Military Academy West Point, NY United States; ^4^ University of Arkansas for Medical Sciences Little Rock, AR United States

**Keywords:** pregnancy, gestational weight gain, lifestyle modification, intervention

## Abstract

**Background:**

Two-thirds of pregnant women exceed gestational weight gain (GWG) recommendations. Because excess GWG is associated with adverse outcomes for mother and child, development of scalable and cost-effective approaches to deliver intensive lifestyle programs during pregnancy is urgent.

**Objective:**

The aim of this study was to decrease the proportion of women who exceed the Institute of Medicine (IOM) 2009 GWG guidelines.

**Methods:**

In a parallel-arm randomized controlled trial, 54 pregnant women (age 18-40 years) who were overweight (n=25) or obese (n=29) were enrolled to test whether an intensive lifestyle intervention (called SmartMoms) decreased the proportion of women with excess GWG, defined as exceeding the 2009 IOM guidelines, compared to no intervention (usual care group). The SmartMoms intervention was delivered through mobile phone (remote group) or in a traditional in-person, clinic-based setting (in-person group), and included a personalized dietary intake prescription, self-monitoring weight against a personalized weight graph, activity tracking with a pedometer, receipt of health information, and continuous personalized feedback from counselors.

**Results:**

A significantly smaller proportion of women exceeded the IOM 2009 GWG guidelines in the SmartMoms intervention groups (in-person: 56%, 10/18; remote: 58%, 11/19) compared to usual care (85%, 11/13; *P*=.02). The remote intervention was a lower cost to participants (mean US $97, SD $6 vs mean US $347, SD $40 per participant; *P*<.001) and clinics (US $215 vs US $419 per participant) and with increased intervention adherence (76.5% vs 60.8%; *P*=.049).

**Conclusions:**

An intensive lifestyle intervention for GWG can be effectively delivered via a mobile phone, which is both cost-effective and scalable.

**Trial Registration:**

Clinicaltrials.gov NCT01610752; https://clinicaltrials.gov/ct2/show/NCT01610752 (Archived by WebCite at http://www.webcitation.org/6sarNB4iW)

## Introduction

More than two-thirds of pregnant women exceed the Institute of Medicine (IOM) 2009 gestational weight gain (GWG) recommendations [1]. According to weight management guidelines for obesity treatment [2], prenatal care should provide an ideal clinical framework for treatment delivery with frequent visits, weight recording, an established definition of acceptable weight gain, and opportunities for in-person counseling. However, despite several efforts to prevent excessive GWG in clinical trials, it remains unclear if lifestyle interventions can be efficacious, particularly in women with overweight or obesity [3,4]. Poor effectiveness in these trials is explained by intervention designs that fail to take advantage of the entire prenatal care continuum because program initiation is often delayed until mid or late gestation and weight management counseling and intervention are limited to one or two in-person sessions [3]. As more patients have access to mobile phones and 67% of pregnant women subscribe to electronic health information delivery during pregnancy [5], eHealth interventions designed to target healthy weight gain provide an opportunity for high-intensity and cost-effective interventions to be delivered to all patients throughout prenatal care. The aim of this study was to test whether a personalized gestational weight management program (SmartMoms) delivered in-person or via an intensity-matched mobile phone app could decrease the proportion of women with overweight and obesity that exceed the IOM 2009 guidelines for GWG by 25%.

## Methods

This study targeted overweight and obese (body mass index [BMI] 25.0-39.9 kg/m^2^) women aged 18 to 40 years expecting a singleton pregnancy in their first trimester. Women with a known fetal anomaly, hypertension (systolic >160 mm Hg or diastolic >90 mm Hg), history of or current psychotic or eating disorder, human immunodeficiency virus, preexisting diabetes (self-report or determined by glycated hemoglobin A_1c_ and/or 75 g oral glucose tolerance test in the first trimester), or with contraindications to exercise (by PARmed-X and American College of Obstetricians and Gynecologists committee opinion #67 [6]) were excluded. With support of local obstetricians, participants were recruited from brochures placed in various clinics and by study staff during the patients’ first prenatal appointment [7]. Participants were randomized by unblinded intervention staff equally to one of three groups between 10.4 to 13.6 weeks of gestation: (1) no intervention (usual care group), (2) receipt of the SmartMoms intervention in-person (in-person group), or (3) receipt of the SmartMoms intervention via mobile phone (remote group), with randomization stratified by pregravid BMI. The block randomization schedule and sealed numbered randomization envelopes were prepared by the biostatistician. Usual care (control) participants were under the usual care of their obstetrician and did not receive weight management services from the intervention team. The SmartMoms intervention was designed to assist an expectant mother in gaining weight within the recommended 2009 IOM guidelines for her respective BMI class. SmartMoms is grounded in the ability to objectively quantify dietary adherence to an energy intake prescription based on measured body weight and to provide patients with data-driven feedback about their energy intake [8-12]. SmartMoms participants received dietary intake advice, exercise advice, and a weight graph created from the dynamic GWG models to determine the trimester-specific increase in energy intake required by each participant to adhere to the IOM GWG recommendations [13]. To promote these lifestyle changes, participants received a structured intervention that consisted of 18 lessons and behavior modification strategies. SmartMoms participants received behavior modification counseling weekly between weeks 13 and 24 of gestation and biweekly from week 25 until delivery. Importantly, the content of the lesson materials were identical and only the mode of delivery differed between the two intervention groups. Participants in both the intervention groups were provided with a wireless Internet-connected bathroom scale and a pedometer (in-person group: Omron Healthcare, Lake Forest, IL, USA; remote group: Fitbit Zip, FitBit, San Francisco, CA, USA) to self-monitor body weight and step counts daily. The mobile phone app is similar to a virtual weight management system described for weight loss in which body weight and daily steps are automatically transmitted in real time to personalized charts [14]. The SmartMoms intervention includes an IOM 2009 GWG weight graph personalized for each patient and behavioral modification tools including daily self-monitoring of weight, dietary intake, and physical activity [14]. SmartMoms participants were provided with an individualized calorie intake above their estimated prepregnancy energy requirement [13] or energy gap represented by an ideal weight gain zone [15], and were coached how to adjust energy intake and/or physical activity to adhere to the IOM 2009 GWG guidelines. The in-person group tracked step counts with pen and paper, and the IOM weight graph was reviewed in hard copy during counseling sessions with interventionists.

Clinic assessments were performed by certified staff who were blinded to group assignment. Maternal weight was measured fasting and in a hospital gown. Total GWG and GWG per week were calculated between the initial (10-13 weeks) and final (35-36 weeks) study visits. GWG per week was used to calculate the proportion of women based on prepregnancy BMI with recommended or excessive GWG per the 2009 IOM GWG guidelines [16].

Adherence to the SmartMoms intervention was defined as the percentage of days participants weighed and recorded step counts in comparison to the expected number of days. Study economics, including costs incurred for travel to and from treatment sessions and time spent with the counselor while accounting for session attendance and intervention adherence, were calculated for each participant. The clinic economics included cost of interventionist time (training, session preparation, participant contacts, routine staff meetings) and equipment (scale, pedometer) cost.

Statistical analyses were completed using SAS/STAT version 9.4 software of the SAS System for Windows (Cary, NC, USA). Sample size was based on the hypothesis that the proportion of overweight and obese pregnant women in the usual care group exceeding IOM guidelines for GWG would be 58% and that lowering this proportion by at least 25% would be clinically significant. Intention-to-treat comparison of continuous variables (eg, GWG, birth weight) between the three treatment groups used one-way analysis of variance with post hoc pairwise intervention group comparisons. Comparisons of categorical variables (eg, prevalence of excess GWG) between the three treatment groups used Fisher exact test. Equality of adherence to IOM GWG guidelines was tested through one-sided z tests for proportions. Finally, differences in study costs and intervention adherence were assessed through two-sample *t* tests. All tests were performed with significance level alpha=.05, and findings were considered significant when *P*<alpha. Data are reported as least square (LS) mean and standard error (SE) unless otherwise noted.

## Results

Recruitment of participants from community clinics from February 1, 2013 to April 14, 2014, yielded three groups of pregnant women who were similar (Table 1). The majority of participants were white and nulliparous or primiparous. No study-related serious adverse events were reported.

### Gestational Weight Gain and Guideline Adherence

**Figure 1:**
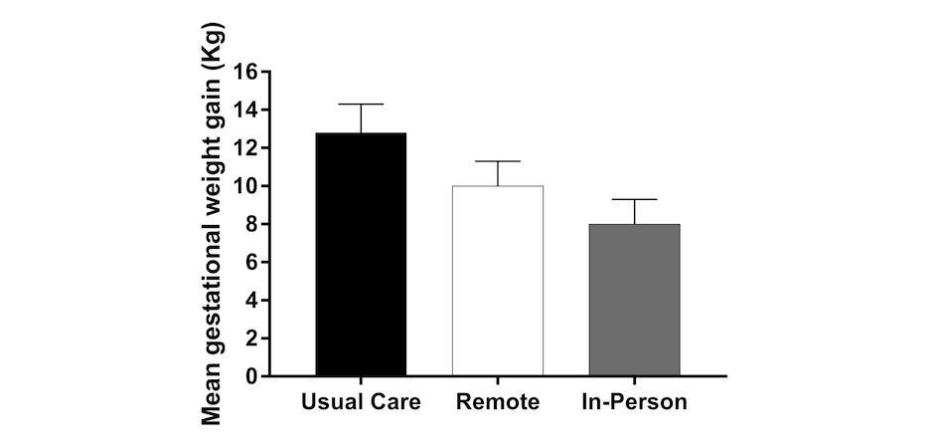
Mean gestational weight gain (kg) for women in the usual care, remote, and in-person groups. The whiskers represent standard error.

**Figure 2:**
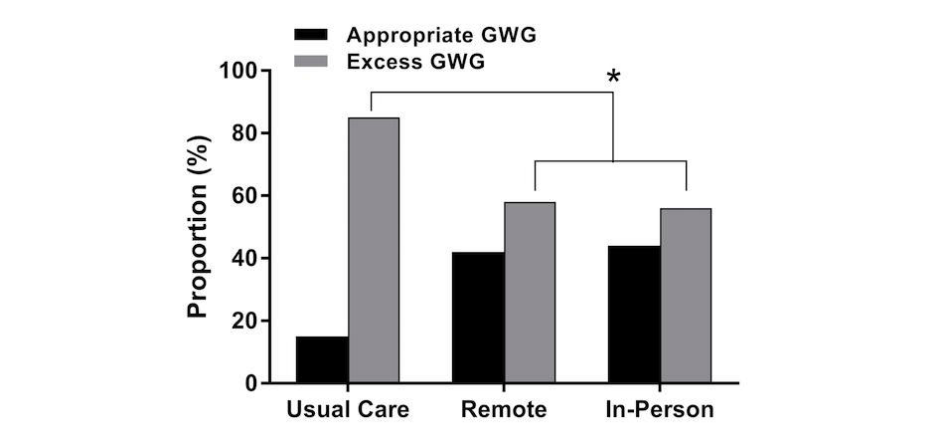
Proportion of women in the usual care, remote, and in-person groups who had appropriate and excess gestational weight gain (GWG) based on the IOM 2009 guidelines. *The SmartMoms intervention (in-person and remote groups combined) was effective at reducing GWG.

The SmartMoms intervention (in-person and remote groups combined) was effective at reducing GWG in overweight and obese pregnant women (usual care: LS mean 12.8, SE 1.5 kg; SmartMoms: LS mean 9.2, SE 0.9 kg; *P*=.04). The in-person group gained significantly less total weight (Figure 1) during pregnancy than the usual care group (LS mean 8.0, SE 1.3 kg vs LS mean 12.8, SE 1.5 kg; *P*=.04) and weight gain in the remote group was equivalent to the in-person group (LS mean 10.0, SE 1.3 kg; *P*=.04 equivalence) and modestly lower than weight gain with usual care (LS mean 10.0, SE 1.2 kg vs LS mean 12.8, SE 1.5 kg; *P*=.07). Compared to usual care, the rate of GWG was significantly lower in the in-person group (LS mean 0.49, SE 0.06 kg/week vs LS mean 0.31, SE 0.05 kg/week; *P*=.01) and the rate of GWG in the in-person group was equivalent to the remote group (LS mean 0.39, SE 0.05 kg/week; *P*=.04) within 200 grams of weight gained per week. The proportion of women with excess GWG (Figure 2) was significantly lower in the in-person (56%, 10/18; *P*=.03) and remote groups (58%, 11/19; *P*=.04) compared to usual care (84.6%, 11/13).

**Table 1:** Baseline characteristics by treatment group (N=54).

Characteristic		Usual care (n=17)	In-person (n=18)	Remote (n=19)	*P*^a^
Age (years), mean (SD)		29.5 (5.1)	29.2 (4.8)	29.0 (4.2)	.96
**Race, n (%)**					.24
	Black	6 (35)	5 (28)	2 (11)	
	White	11 (65)	11 (61)	16 (84)	
	Other	0 (0)	2 (11)	1 (5)	
Weight (kg), mean (SD)		86.2 (12)	83.0 (12)	81.1 (13)	.45
Parity, n (%)		0.6 (1)	0.7 (1)	0.9 (1)	.54
**Pregravid BMI group, n (%)**					.84
	Overweight (25.0-29.9 kg/m^2^)	9 (53)	8 (44)	8 (42)	
	Obese (30.0-40.0 kg/m^2^)	8 (47)	10 (56)	11 (58)	
**Family income per year (US$), n (%)**					.50
	<$5000-$39,999	7 (41)	9 (50)	7 (37)	
	$40,000-$99,999	5 (29)	7 (39)	5 (26)	
	≥$100,000	5 (29)	2 (11)	7 (37)	
**Education, n (%)**					.24
	Some high school	0 (0)	1 (6)	0 (0)	
	High school diploma/GED/1-3 years of college, business, or technical school	7 (41)	5 (28)	6 (32)	
	College degree	4 (24)	7 (39)	11 (58)	
	Postgraduate work	6 (35)	5 (28)	2 (11)	

^a^*P* values were derived from ANOVA for continuous variables and Fisher exact test for categorical variables.

### Intervention Adherence and Study Economics

**Figure 3:**
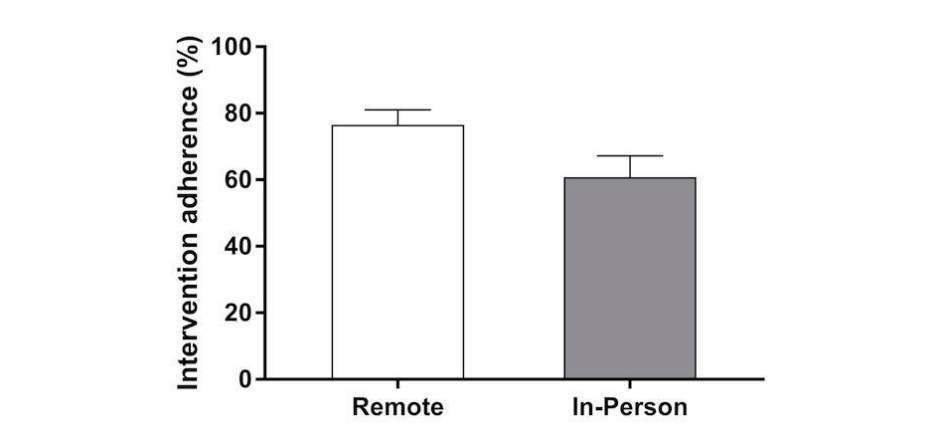
Intervention adherence for the remote and in-person groups. The whiskers represent standard error.

**Figure 4:**
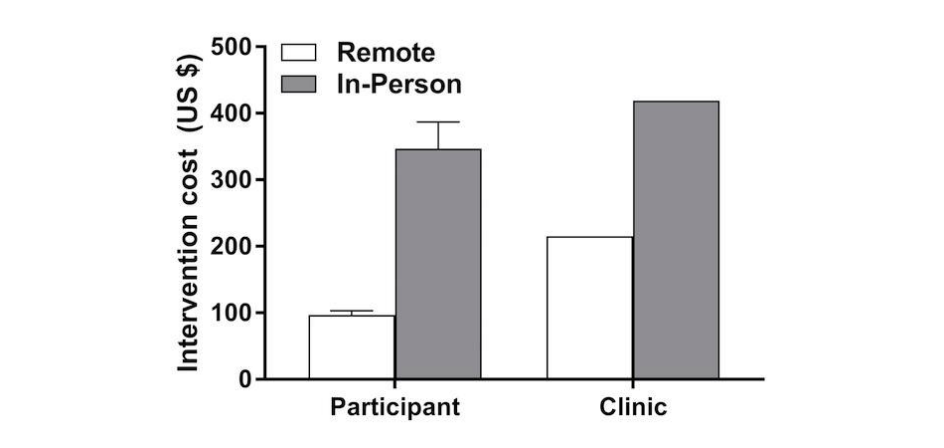
Mean intervention cost (US $) for participants and clinics for the remote and in-person groups. The whiskers represent standard error. The intervention cost incurred by the clinic was fixed.

The in-person group recorded weight and step data (weight: mean 57.2%, SD 33.8%; step: mean 44.5%, SD 33.3%) less often than the remote group (weight: mean 71.2%, SD 24.1%; step: mean 72.5, SD 29.0%) and the in-person group attended mean 78% (SD 39%) of planned behavioral sessions. Therefore, overall intervention adherence (Figure 3) was greater in the remote group than the in-person group (76.5% vs 60.8%; *P*=.049). The intervention cost (Figure 4) to a participant in the remote group was 3.5 times less than the cost for a participant in the in-person group (mean US $97, SD $6 vs mean US $347, SD $40 per participant; *P*<.001). Similarly, the clinic cost to deploy the remote intervention was 50% less than the cost to deploy the in-person intervention (US $215 vs US $419 per participant).

## Discussion

Lifestyle interventions to improve adherence to the IOM GWG recommendations have had modest success in reducing GWG [4,17] and little impact reducing the incidence of excess GWG [18]. The greatest success has been with a recommendation of caloric restriction [19]; however, due to popular beliefs such as the need to “eat for two” [20], caloric restriction is not widely accepted among patients, practitioners, or their support systems. Albeit in a small sample, we attribute the success of the SmartMoms intervention to its early initiation (13 weeks gestation) and intervention intensiveness being commensurate with weight management treatment for nonpregnant individuals including self-monitoring with timely feedback, a dietary prescription to foster optimal weight change, and receipt of structured behavior change intervention through delivery of 18 lessons over a 24-week interval beginning at the second trimester.

When deployed remotely through a mobile phone, the SmartMoms intervention was just as effective at reducing the proportion of excess GWG when delivered in-person; however, it was found to be at least 50% more economical for patients and providers with a higher level of patient engagement or adherence. This eHealth intervention, including the provision of a personalized IOM GWG weight graph through Internet-connected devices, easily disseminates supportive health information to patients, and remote patient communication provides an ideal framework for integration into an electronic health record system. Using estimates of interventionist time recorded throughout the study, it is estimated that approximately 30 to 50 new patients per clinician per month could be monitored simultaneously through the remote program by a single health care provider, such as a dietician or lifestyle coach, for universal delivery to all patients within a clinical practice. Similar telehealth services are covered by health care insurance companies, including Medicaid, and are already used by health care facilities across the United States such as in the Veterans Affairs Hospital System for management of chronic health conditions [21]. The SmartMoms mobile phone intervention tested on community-based obstetrical patients could easily be integrated into standard clinical practice, thereby improving access to effective and efficient health care for millions of American women throughout the entire prenatal care continuum.

## Abbreviations

BMI: body mass index
GWG: gestational weight gain
IOM: Institute of Medicine

## Appendix

Multimedia Appendix 1 CONSORT-EHEALTH checklist (v1.6.1).
